# Supplementary material for: Dosimetry of [212Pb]VMT01, a MC1R-Targeted Alpha Therapeutic Compound, and Effect of Free 208Tl on Tissue Absorbed Doses
Source: Molecules. 2022 Sep 8;27(18):5831. doi: 10.3390/molecules27185831 (PMC9504749; doi:10.3390/molecules27185831)
Supplement: Supplementary file 1 [file molecules-27-05831-s001.zip › molecules-1873500-supplementary.pdf]

SUPPLEMENTARY MATERIAL - Dosimetry of [^{212}Pb]VMT01, a MC1R-targeted Alpha Therapeutic Compound, and Effect of Free ^{208}Tl on Tissue Absorbed Doses - Orcutt KD *et al*

Supplemental Table S1. Organ/tissue activity concentrations (%ID/g) of [203Pb]VMT01 in female CD-1 IGS naïve mice.

Organ		Time (hr)						
		0.5	1	2	4	6	24	55
Adrenals	Mean	6.42×10^{-1}	3.46×10^{-1}	0.00×10^{0}	6.77×10^{-2}	0.00×10^{0}	0.00×10^{0}	0.00×10^{0}
	SEM	1.36×10^{-1}	1.29×10^{-1}	0.00×10^{0}	2.98×10^{-2}	0.00×10^{0}	0.00×10^{0}	0.00×10^{0}
Bladder wall	Mean	5.88×10^{0}	1.16×10^{0}	4.92×10^{-1}	1.52×10^{-1}	1.33×10^{-2}	0.00×10^{0}	3.05×10^{-3}
	SEM	2.87×10^{0}	5.61×10^{-1}	3.10×10^{-1}	5.97×10^{-2}	7.84×10^{-3}	0.00×10^{0}	3.05×10^{-3}
Blood (whole)	Mean	1.18×10^{0}	3.35×10^{-1}	2.14×10^{-2}	4.24×10^{-2}	2.23×10^{-3}	0.00×10^{0}	3.61×10^{-2}
	SEM	2.51×10^{-1}	1.43×10^{-1}	9.59×10^{-3}	2.69×10^{-3}	2.23×10^{-3}	0.00×10^{0}	7.11×10^{-3}
Carcass	Mean	1.22×10^{0}	4.31×10^{-1}	-	1.57×10^{-1}	-	-	-
	SEM	2.11×10^{-1}	1.27×10^{-1}	-	3.38×10^{-2}	-	-	-
Bone	Mean	7.17×10^{-1}	1.99×10^{-1}	3.14×10^{-2}	2.00×10^{-1}	3.13×10^{-2}	5.91×10^{-3}	2.96×10^{-3}
	SEM	1.87×10^{-1}	5.14×10^{-2}	2.19×10^{-2}	6.58×10^{-2}	2.02×10^{-2}	3.96×10^{-3}	2.96×10^{-3}
Bone Marrow	Mean	6.85×10^{-2}	1.59×10^{-1}	0.00×10^{0}	9.94×10^{-2}	0.00×10^{0}	0.00×10^{0}	0.00×10^{0}
	SEM	6.85×10^{-2}	1.59×10^{-1}	0.00×10^{0}	9.94×10^{-2}	0.00×10^{0}	0.00×10^{0}	0.00×10^{0}
Brain	Mean	4.61×10^{-2}	1.68×10^{-2}	0.00×10^{0}	4.08×10^{-3}	2.52×10^{-4}	0.00×10^{0}	4.75×10^{-4}
	SEM	5.43×10^{-3}	4.48×10^{-3}	0.00×10^{0}	1.20×10^{-3}	2.52×10^{-4}	0.00×10^{0}	4.75×10^{-4}
Cecum	Mean	2.66×10^{-1}	9.60×10^{-2}	2.15×10^{-1}	5.46×10^{-1}	1.77×10^{0}	4.74×10^{-1}	1.18×10^{-1}
	SEM	5.71×10^{-2}	2.06×10^{-2}	5.65×10^{-2}	2.38×10^{-1}	5.42×10^{-1}	1.63×10^{-1}	5.85×10^{-2}
Eyes	Mean	4.78×10^{-1}	1.33×10^{-1}	4.04×10^{-3}	6.29×10^{-2}	2.89×10^{-3}	0.00×10^{0}	3.29×10^{-3}
	SEM	1.69×10^{-1}	1.42×10^{-2}	2.21×10^{-3}	1.98×10^{-2}	2.89×10^{-3}	0.00×10^{0}	3.29×10^{-3}
Gallbladder	Mean	5.28×10^{-1}	1.06×10^{-1}	0.00×10^{0}	1.27×10^{-1}	0.00×10^{0}	0.00×10^{0}	2.69×10^{-2}
	SEM	2.76×10^{-1}	3.85×10^{-2}	0.00×10^{0}	6.58×10^{-2}	0.00×10^{0}	0.00×10^{0}	2.69×10^{-2}
Heart	Mean	5.91×10^{-1}	1.67×10^{-1}	1.98×10^{-2}	3.31×10^{-2}	6.42×10^{-3}	7.64×10^{-3}	1.65×10^{-3}
	SEM	1.22×10^{-1}	6.38×10^{-2}	2.03×10^{-3}	5.75×10^{-3}	2.95×10^{-3}	4.63×10^{-3}	1.09×10^{-3}
Kidneys	Mean	6.24×10^{0}	4.89×10^{0}	4.28×10^{0}	4.51×10^{0}	4.65×10^{0}	2.72×10^{0}	1.09×10^{0}
	SEM	3.51×10^{-1}	2.50×10^{-1}	4.45×10^{-1}	6.62×10^{-1}	4.28×10^{-1}	4.54×10^{-1}	1.21×10^{-1}
Large intestines	Mean	8.01×10^{-1}	4.60×10^{-1}	1.28×10^{-1}	2.85×10^{-1}	3.30×10^{-1}	1.50×10^{-1}	4.28×10^{-2}
	SEM	1.55×10^{-1}	1.23×10^{-1}	4.26×10^{-2}	9.82×10^{-2}	1.40×10^{-1}	5.87×10^{-2}	1.69×10^{-2}
Large intestines contents	Mean	3.26×10^{-2}	3.27×10^{-2}	7.47×10^{-2}	1.26×10^{0}	3.03×10^{0}	1.58×10^{0}	1.78×10^{-1}
	SEM	1.43×10^{-2}	1.17×10^{-2}	4.01×10^{-2}	7.62×10^{-1}	1.18×10^{0}	7.13×10^{-1}	1.08×10^{-1}
Liver	Mean	6.88×10^{-1}	5.16×10^{-1}	3.33×10^{-1}	4.96×10^{-1}	3.50×10^{-1}	2.40×10^{-1}	2.13×10^{-1}
	SEM	7.53×10^{-2}	2.48×10^{-2}	2.08×10^{-2}	6.22×10^{-2}	3.93×10^{-2}	2.42×10^{-2}	3.27×10^{-2}
Lungs	Mean	1.39×10^{0}	8.61×10^{-1}	1.06×10^{-1}	1.13×10^{-1}	6.10×10^{-2}	1.44×10^{-2}	1.28×10^{-1}
	SEM	2.29×10^{-1}	1.95×10^{-1}	1.44×10^{-2}	2.28×10^{-2}	2.62×10^{-2}	7.57×10^{-3}	8.00×10^{-2}
Muscle	Mean	3.22×10^{-1}	1.12×10^{-1}	4.06×10^{-3}	5.07×10^{-2}	5.94×10^{-2}	0.00×10^{0}	0.00×10^{0}
	SEM	5.22×10^{-2}	4.58×10^{-2}	4.06×10^{-3}	2.15×10^{-2}	5.94×10^{-2}	0.00×10^{0}	0.00×10^{0}
Ovaries	Mean	5.27×10^{-1}	2.47×10^{-1}	2.84×10^{-2}	5.39×10^{-2}	1.05×10^{-2}	3.91×10^{-2}	2.49×10^{-2}
	SEM	3.54×10^{-2}	7.41×10^{-2}	1.80×10^{-2}	1.05×10^{-2}	6.52×10^{-3}	1.96×10^{-2}	7.79×10^{-3}
Pancreas	Mean	3.87×10^{-1}	1.28×10^{-1}	1.53×10^{-2}	5.30×10^{-2}	6.59×10^{-3}	4.10×10^{-3}	1.60×10^{-3}
	SEM	7.93×10^{-2}	4.15×10^{-2}	7.47×10^{-3}	1.47×10^{-2}	3.90×10^{-3}	4.10×10^{-3}	1.60×10^{-3}
Skin	Mean	3.18×10^{0}	5.60×10^{-1}	3.46×10^{-1}	3.22×10^{-1}	1.87×10^{-1}	5.12×10^{-2}	2.04×10^{-2}
	SEM	1.02×10^{0}	1.51×10^{-1}	8.25×10^{-2}	1.26×10^{-1}	5.22×10^{-2}	1.01×10^{-2}	1.16×10^{-2}
Small intestines	Mean	5.50×10^{-1}	2.58×10^{-1}	4.55×10^{-1}	1.01×10^{-1}	2.90×10^{-1}	6.01×10^{-2}	2.28×10^{-2}
	SEM	1.05×10^{-1}	6.73×10^{-2}	7.85×10^{-2}	8.59×10^{-3}	8.40×10^{-2}	1.94×10^{-2}	1.18×10^{-3}
Small intestines Contents	Mean	1.94×10^{-1}	1.83×10^{-1}	2.52×10^{0}	1.73×10^{-1}	2.10×10^{0}	3.48×10^{-1}	1.74×10^{-1}
	SEM	2.49×10^{-2}	1.25×10^{-2}	8.66×10^{-1}	4.38×10^{-2}	8.71×10^{-1}	1.96×10^{-1}	3.68×10^{-2}
Spleen	Mean	4.43×10^{-1}	3.77×10^{-1}	1.21×10^{-1}	3.94×10^{-1}	8.03×10^{-2}	6.21×10^{-2}	1.34×10^{-1}
	SEM	6.59×10^{-2}	3.84×10^{-2}	2.94×10^{-2}	1.59×10^{-1}	1.91×10^{-2}	1.94×10^{-2}	5.75×10^{-2}
Stomach Contents	Mean	1.33×10^{-1}	4.39×10^{-2}	2.06×10^{0}	3.86×10^{-2}	8.39×10^{-1}	1.09×10^{-1}	3.72×10^{-2}
	SEM	2.11×10^{-2}	2.14×10^{-3}	5.91×10^{-1}	1.57×10^{-2}	6.40×10^{-1}	8.46×10^{-2}	1.34×10^{-2}
Stomach Wall	Mean	7.04×10^{-1}	3.16×10^{-1}	5.57×10^{-1}	1.44×10^{-1}	3.43×10^{-1}	1.08×10^{-1}	4.02×10^{-2}
	SEM	1.42×10^{-1}	4.06×10^{-2}	1.91×10^{-1}	2.07×10^{-2}	1.55×10^{-1}	3.58×10^{-2}	6.82×10^{-3}
Thymus	Mean	4.84×10^{-1}	1.38×10^{-1}	2.16×10^{-2}	6.27×10^{-2}	1.81×10^{-3}	0.00×10^{0}	9.73×10^{-3}
	SEM	9.42×10^{-2}	5.76×10^{-2}	2.16×10^{-2}	2.75×10^{-2}	1.81×10^{-3}	0.00×10^{0}	9.73×10^{-3}
Thyroid	Mean	1.06×10^{0}	4.29×10^{-1}	0.00×10^{0}	1.91×10^{-1}	0.00×10^{0}	0.00×10^{0}	3.80×10^{-2}
	SEM	2.14×10^{-1}	1.87×10^{-1}	0.00×10^{0}	7.74×10^{-2}	0.00×10^{0}	0.00×10^{0}	3.80×10^{-2}

Organ		Time (hr)						
		0.5	1	2	4	6	24	55
Uterus	Mean	1.34×10^{0}	3.32×10^{-1}	1.00×10^{-1}	1.12×10^{-1}	5.36×10^{-2}	2.45×10^{-2}	1.53×10^{-2}
	SEM	2.50×10^{-1}	1.24×10^{-1}	2.28×10^{-2}	2.87×10^{-2}	6.06×10^{-3}	2.12×10^{-2}	4.52×10^{-3}

Supplemental Table S2. Organ/tissue activity concentrations (%ID/g) of [203Pb]VMT01 in male CD-1 IGS naïve mice.

Organ		Time (hr)						
		0.5	1	2	4	6	24	55
Adrenal glands	Mean	1.31×10^{0}	3.56×10^{0}	1.78×10^{-2}	2.10×10^{-1}	1.04×10^{-1}	0.00×10^{0}	0.00×10^{0}
	SEM	7.49×10^{-1}	3.12×10^{0}	1.78×10^{-2}	1.74×10^{-1}	7.66×10^{-2}	0.00×10^{0}	0.00×10^{0}
Bladder wall	Mean	2.64×10^{1}	2.92×10^{1}	7.37×10^{-1}	4.27×10^{0}	3.51×10^{0}	1.52×10^{-2}	0.00×10^{0}
	SEM	1.01×10^{1}	1.59×10^{1}	5.18×10^{-1}	3.09×10^{0}	3.15×10^{0}	6.10×10^{-3}	0.00×10^{0}
Blood whole	Mean	1.37×10^{0}	3.35×10^{-1}	5.85×10^{-2}	5.63×10^{-3}	4.21×10^{-3}	0.00×10^{0}	9.28×10^{-3}
	SEM	3.00×10^{-1}	1.08×10^{-1}	1.05×10^{-2}	2.36×10^{-3}	4.21×10^{-3}	0.00×10^{0}	9.28×10^{-3}
Remainder Body	Mean	8.47×10^{-1}	5.68×10^{-1}	-	1.70×10^{-1}	2.19×10^{-1}	-	-
	SEM	1.05×10^{-1}	8.80×10^{-2}	-	1.06×10^{-2}	3.43×10^{-2}	-	-
Bone	Mean	4.62×10^{-1}	1.64×10^{-1}	1.35×10^{-1}	1.54×10^{-1}	4.76×10^{-1}	6.44×10^{-3}	3.72×10^{-2}
	SEM	1.02×10^{-1}	7.16×10^{-2}	7.79×10^{-2}	2.88×10^{-2}	2.65×10^{-1}	4.19×10^{-3}	3.72×10^{-2}
Bone Marrow	Mean	0.00×10^{0}	0.00×10^{0}	0.00×10^{0}	1.54×10^{-2}	5.25×10^{-4}	0.00×10^{0}	0.00×10^{0}
	SEM	0.00×10^{0}	0.00×10^{0}	0.00×10^{0}	1.54×10^{-2}	5.25×10^{-4}	0.00×10^{0}	0.00×10^{0}
Brain whole	Mean	4.48×10^{-2}	2.25×10^{-2}	2.36×10^{-3}	1.88×10^{-2}	1.18×10^{-2}	0.00×10^{0}	0.00×10^{0}
	SEM	1.06×10^{-2}	6.84×10^{-3}	9.34×10^{-4}	5.41×10^{-3}	5.90×10^{-3}	0.00×10^{0}	0.00×10^{0}
Cecum	Mean	2.68×10^{-1}	1.18×10^{-1}	1.51×10^{-1}	2.33×10^{0}	1.70×10^{0}	1.04×10^{-1}	1.12×10^{0}
	SEM	6.66×10^{-2}	2.48×10^{-2}	3.53×10^{-2}	3.83×10^{-1}	3.90×10^{-1}	2.91×10^{-2}	5.32×10^{-1}
Eyes	Mean	4.03×10^{-1}	1.55×10^{-1}	5.35×10^{-2}	8.41×10^{-2}	4.26×10^{-2}	0.00×10^{0}	0.00×10^{0}
	SEM	7.24×10^{-2}	2.65×10^{-2}	1.30×10^{-2}	1.37×10^{-2}	4.12×10^{-3}	0.00×10^{0}	0.00×10^{0}
Gallbladder	Mean	4.52×10^{-1}	2.68×10^{-2}	0.00×10^{0}	4.79×10^{-2}	3.52×10^{-2}	0.00×10^{0}	0.00×10^{0}
	SEM	2.61×10^{-1}	2.68×10^{-2}	0.00×10^{0}	4.79×10^{-2}	3.52×10^{-2}	0.00×10^{0}	0.00×10^{0}
Heart	Mean	6.78×10^{-1}	1.75×10^{-1}	4.09×10^{-2}	2.04×10^{-2}	2.73×10^{-2}	8.77×10^{-3}	5.16×10^{-3}
	SEM	1.28×10^{-1}	5.52×10^{-2}	7.20×10^{-3}	4.17×10^{-3}	1.51×10^{-2}	4.40×10^{-3}	2.60×10^{-3}
Kidneys	Mean	8.30×10^{0}	6.70×10^{0}	4.78×10^{0}	4.20×10^{0}	3.36×10^{0}	1.30×10^{0}	5.47×10^{-1}
	SEM	1.90×10^{0}	4.78×10^{-1}	6.58×10^{-1}	5.67×10^{-1}	4.98×10^{-1}	2.76×10^{-1}	1.04×10^{-1}
Large intestines	Mean	8.59×10^{-1}	6.70×10^{-1}	1.08×10^{-1}	9.77×10^{-1}	5.53×10^{-1}	4.57×10^{-2}	4.70×10^{-1}
	SEM	2.25×10^{-1}	1.60×10^{-1}	2.48×10^{-2}	5.77×10^{-1}	1.06×10^{-1}	8.69×10^{-3}	2.43×10^{-1}
Large intestines contents	Mean	3.16×10^{-2}	8.36×10^{-2}	1.33×10^{-1}	4.66×10^{0}	4.71×10^{0}	1.91×10^{-1}	2.03×10^{0}
	SEM	6.79×10^{-3}	4.70×10^{-2}	2.83×10^{-2}	1.81×10^{0}	1.09×10^{0}	7.10×10^{-2}	1.09×10^{0}
Liver	Mean	6.36×10^{-1}	3.48×10^{-1}	2.19×10^{-1}	2.68×10^{-1}	2.06×10^{-1}	1.75×10^{-1}	1.02×10^{-1}
	SEM	3.43×10^{-2}	3.59×10^{-2}	4.09×10^{-2}	2.76×10^{-2}	2.26×10^{-2}	3.26×10^{-2}	2.61×10^{-2}
Lungs	Mean	2.15×10^{0}	4.96×10^{-1}	1.42×10^{-1}	1.65×10^{-1}	6.63×10^{-2}	3.25×10^{-2}	1.08×10^{-2}
	SEM	3.80×10^{-1}	1.24×10^{-1}	1.91×10^{-2}	2.42×10^{-2}	1.00×10^{-2}	1.01×10^{-2}	9.87×10^{-3}
Muscle	Mean	3.36×10^{-1}	2.54×10^{-1}	5.39×10^{-2}	4.77×10^{-2}	4.11×10^{-2}	0.00×10^{0}	0.00×10^{0}
	SEM	6.03×10^{-2}	5.76×10^{-2}	1.51×10^{-2}	1.13×10^{-2}	4.98×10^{-3}	0.00×10^{0}	0.00×10^{0}
Pancreas	Mean	4.97×10^{-1}	1.12×10^{-1}	3.02×10^{-2}	4.27×10^{-2}	1.04×10^{-1}	3.11×10^{-3}	0.00×10^{0}
	SEM	6.69×10^{-2}	3.27×10^{-2}	8.78×10^{-3}	6.65×10^{-3}	7.25×10^{-2}	1.65×10^{-3}	0.00×10^{0}
Skin	Mean	1.12×10^{0}	6.63×10^{-1}	6.31×10^{-1}	1.94×10^{-1}	5.03×10^{-1}	2.52×10^{-2}	2.19×10^{-2}
	SEM	1.66×10^{-1}	1.69×10^{-1}	2.76×10^{-1}	2.29×10^{-2}	1.54×10^{-1}	7.91×10^{-3}	9.02×10^{-3}
Small intestines	Mean	6.43×10^{-1}	3.70×10^{-1}	3.46×10^{-1}	4.31×10^{-1}	2.56×10^{-1}	2.78×10^{-2}	2.74×10^{-1}
	SEM	1.09×10^{-1}	3.48×10^{-2}	7.68×10^{-2}	1.48×10^{-1}	3.08×10^{-2}	3.60×10^{-3}	9.38×10^{-2}
Small intestines Contents	Mean	4.45×10^{-1}	6.83×10^{-1}	1.92×10^{0}	2.16×10^{0}	9.12×10^{-1}	4.01×10^{-2}	1.95×10^{0}
	SEM	1.47×10^{-1}	1.48×10^{-1}	3.40×10^{-1}	4.17×10^{-1}	2.87×10^{-1}	8.01×10^{-3}	6.71×10^{-1}
Spleen	Mean	6.32×10^{-1}	1.94×10^{-1}	8.03×10^{-2}	1.83×10^{-1}	1.37×10^{-1}	7.47×10^{-2}	2.41×10^{-2}
	SEM	3.69×10^{-2}	2.32×10^{-2}	2.16×10^{-2}	3.14×10^{-2}	3.68×10^{-2}	3.25×10^{-3}	2.41×10^{-2}
Stomach Contents	Mean	6.54×10^{-1}	5.63×10^{-1}	2.49×10^{0}	5.74×10^{-1}	3.05×10^{-1}	1.59×10^{-2}	4.27×10^{-1}
	SEM	2.85×10^{-1}	1.55×10^{-1}	6.86×10^{-1}	6.93×10^{-2}	3.86×10^{-2}	2.20×10^{-3}	2.60×10^{-1}
Stomach Wall	Mean	8.56×10^{-1}	4.42×10^{-1}	8.56×10^{-1}	3.04×10^{-1}	1.94×10^{-1}	7.88×10^{-2}	1.35×10^{-1}
	SEM	1.54×10^{-1}	6.77×10^{-2}	2.04×10^{-1}	6.51×10^{-2}	2.68×10^{-2}	3.15×10^{-3}	4.64×10^{-2}
Testes	Mean	4.12×10^{-1}	3.41×10^{-1}	1.51×10^{-1}	1.06×10^{-1}	7.57×10^{-2}	1.51×10^{-2}	4.19×10^{-3}
	SEM	6.47×10^{-2}	1.62×10^{-1}	7.81×10^{-2}	7.30×10^{-2}	3.58×10^{-2}	1.10×10^{-3}	2.54×10^{-3}
Thymus	Mean	8.23×10^{-1}	1.36×10^{-1}	4.55×10^{-2}	1.35×10^{-1}	1.90×10^{-2}	0.00×10^{0}	0.00×10^{0}
	SEM	1.32×10^{-1}	3.50×10^{-2}	1.17×10^{-2}	7.68×10^{-2}	8.70×10^{-3}	0.00×10^{0}	0.00×10^{0}

Organ		Time (hr)						
		**0.5**	**1**	**2**	**4**	**6**	**24**	**55**
Thyroid	Mean	1.33×10^{0}	1.47×10^{-1}	6.36×10^{-2}	2.51×10^{-1}	2.51×10^{-1}	0.00×10^{0}	5.82×10^{-2}
	SEM	4.63×10^{-1}	8.11×10^{-2}	2.16×10^{-2}	5.13×10^{-2}	2.38×10^{-1}	0.00×10^{0}	5.82×10^{-2}
